# Toxicity of formaldehyde, and its role in the formation of harmful and aromatic compounds during food processing

**DOI:** 10.1016/j.fochx.2025.102225

**Published:** 2025-01-29

**Authors:** Xiaoyan Sun, Chunmin Yang, Weiyue Zhang, Jie Zheng, Juanying Ou, Shiyi Ou

**Affiliations:** a Engineering Technology Research Center for Health and Nutritional Baked Foods, Guangzhou College of Technology and Business, Guangzhou 510850, China; b Guangdong-Hong Kong Joint Innovation Platform for the Safety of Bakery Products, Jinan University, Guangzhou 510632, China

**Keywords:** Formaldehyde, Derived toxin, Derived aromas, Pathways, Food processing

## Abstract

Formaldehyde is a highly reactive compound known to pose several health risks, including carcinogenic, neurotoxic, reproductive, allergic, immunological, genetic, and respiratory toxicity. While its free concentration in processed foods is typically low even it can be formed through various biochemical and chemical pathways in foods. This study aims to investigate the fate of formaldehyde in food processing from two key perspectives: (1) its role in the formation of other harmful compounds, such as heterocyclic aromatic amines, methylimidazole, advanced glycation end-products, and N-nitrosamines, and (2) its potential to contribute to the generation of aromatic compounds, including oxygen-, sulfur-, and nitrogen-containing heterocyclic aromas. This review provides insights that may help food scientists develop strategies to mitigate formaldehyde's harmful effects while potentially harnessing its role in producing beneficial aromatic compounds.

## Highlights

• Formaldehyde (FA) is a toxin generated in foods via biochemical and chemical pathways.
• FA participates in the formation of other toxins during food processing.
• FA still contributes to heterocyclic aromas formation.
• Diverting the harmful to beneficial effects of FA is a potential strategy for its control.

## 1 Introduction

Formaldehyde is the simplest and smallest aldehyde, characterized by its high reactivity and toxicity. Acute exposure to formaldehyde causes irritation and injury to the skin, eyes, and upper respiratory mucosa. Chronic exposure is linked to a range of toxic effects, including genetic toxicity, neurotoxicity, reproductive toxicity, and respiratory damage (Li et al., 2023). Long-term, low-dose formaldehyde inhalation has been shown to impair learning and memory functions, and induce pathological changes in the lungs and liver (Cheng et al., 2016). Importantly, formaldehyde has been classified as a Group 1 carcinogen by the International Agency for Research on Cancer (IARC) due to its association with nasopharyngeal cancer and leukemia (European Food Safety Authority, 2014). The U.S. Environmental Protection Agency (EPA) recommended a permissible exposure limit of 0.75 ppm for average daily exposure (Kangarlou et al., 2023).

Formaldehyde occurs as a ubiquitous toxin in both raw and processed food materials and can be produced via the biochemical and chemical pathways (Li et al., 2023). It is a critical metabolic intermediate produced physiologically in all cells of the body (European Food Safety Authority, 2014). In raw food materials, formaldehyde is generated primarily through the oxidation of methanol (Xiao & He, 2017), a toxic agent that can induce nausea, vomiting, abdominal pain, and central nervous system depression, vision problems, liver impact, Alzheimer's, and Parkinson's (Abubakar et al., 2023; Nekoukar et al., 2021). Methanol can be derived from the demethylation of DNA, RNA, and histones in all living organisms, as well as from pectin in plants (Dorokhov et al., 2018; Toh et al., 2020). Formaldehyde is also produced via various metabolic processes, including methylamine deamination, glycine oxidation, and the glycine cleavage system in animals, as well as the oxidation of trimethylamine in fish and the decomposition of lentinic acid in shiitake mushrooms (Reingruber & Pontel, 2018). Thus, FA exists in a wide range of food raw materials (Li et al., 2023), and increases during their preservation, even frozen preservation (Table 1). In processed foods, formaldehyde can be produced through the reaction of carbohydrates, lipids, ascorbic acid, and amino acids, particularly during the cleavage of α-dicarbonyl compounds or their Strecker degradation (Li et al., 2023).

**Table 1:** Changes in formaldehyde during the preservation, thermal processing, and fermentation of foods.

Treatment of Foods	Changes in FA	Consequence	References
Preservation	FA increased in fruits (banana, mandarin) during preservation, and increased by several folds after frozen preservation of fish and meats.	Induces protein denaturation and influences the muscle texture.	Bianchi et al., 2007; Jinadasa et al., 2022; Nowshad et al., 2018
Fermentation	Microorganisms induce FA formation in fermented foods, such as yogurt, cheese, wine, whisky, beer	The production of formaldehyde limits the consumption of fermented products, especially fermented plant products	Jeong et al., 2015; Zhang, Zhang, & Mujumdar, 2021
Thermal processing	Cooking and frying decreased FA content in beef, poultry, fish, and shiitake mushroom, etc.	Thermal processing actually generates FA. Reduction of FA in these foods indicated that FA is converted to other compounds.	Bianchi et al., 2007; Mason et al., 2004; Nowshad et al., 2018,

Despite the known health risks of formaldehyde, the levels of free formaldehyde in foods are often overlooked, mainly due to its rapid metabolism in the human body. After ingestion, formaldehyde is quickly converted to formic acid by dehydrogenase enzymes (Dhareshwar & Stella, 2008). According to the European Food Safety Authority (2014), the contribution of formaldehyde from food sources was considered negligible dueto its high turnover (878–1310 mg/kg body weight per day) in the body. In line with this, most of the formaldehyde formed is eliminated rapidly during thermal processing of food (Table 1). However, the specific mechanisms by which formaldehyde disappears from foods remain poorly understood.

In addition to discussing the toxic effects of formaldehyde, this review focused on its fate during food processing. Specifically, it examines two key aspects. The first one is the formation of formaldehyde-derived toxins, such as heterocyclic aromatic amines, methylimidazole, advanced glycation end products, N-nitrosamines, cross-linking adducts, acrolein, and acrylamide. The second one is the formation of formaldehyde-derived flavor compounds, including oxygen-, sulfur-, and nitrogen-containing heterocyclic aromas. A comprehensive understanding of these processes, from both positive and negative perspectives, could assist food scientists in developing strategies to mitigate formaldehyde's harmful effects while potentially exploiting its role in the creation of desirable aromatic compounds.

## 2 Toxic effects of formaldehyde

### 2.1 Acute toxicity

Acute inhalation exposure to formaldehyde (in the form of formalin) can affect multiple organs, including the eyes, nose, throat, skin, and both the upper and lower respiratory tracts (Hagos et al., 2018). Exposure to formaldehyde causes irritation and corrosion of the eyes, leading to symptoms such as burning sensations, lacrimation, conjunctivitis, corneal clouding, and, in severe cases, loss of vision. It also irritates the upper respiratory system, potentially triggering bronchial asthma, pneumonia, pulmonary edema, and bronchospasm (Hagos et al., 2018). Furthermore, acute short-term exposure to formaldehyde has been found to be associated with a rapid decline in vascular function in the upper extremities and an increase in oxidative stress (Augenreich et al., 2020).

Oral exposure to formaldehyde has been shown to induce DNA damage, apoptosis, and central nervous system injury in rabbits (Arici et al., 2014). Elevated levels of formaldehyde in the body can lead to rapid fixation in the blood and acute circulatory disturbances, resulting in damage to nearly all organs in humans. In one case, a woman who died after accidentally ingesting formalin had blood and stomach formaldehyde concentrations of 36.56 mg/kg and 274.48 mg/kg, respectively. These elevated levels of formaldehyde caused rapid fixation in the blood and protein coagulation in tissues. Post-mortem examination revealed severe pathological changes in multiple organs, including congestion in the stomach, liver, kidneys, spleen, and pancreas; edema in the intestines and brain; hemorrhaging in the trachea and lungs; and necrosis of cardiomyocytes (Zhang et al., 2022).

### 2.2 Chronic toxicities

The high reactivity of formaldehyde, combined with its ability to permeate cells, facilitates its interaction with proteins and nucleic acids. As a well-established cross-linking agent, formaldehyde modifies proteins, including nucleoproteins, by reacting with amino groups to form methylol adducts, which are subsequently dehydrated to yield labile Schiff bases. These intermediate compounds can then react with nearby amino acid residues, such as lysine, cysteine, arginine, and tyrosine, leading to both intra- and inter-protein cross-linking (Metz et al., 2004; Tayri-Wilk et al., 2020). The cross-linking of proteins and other macromolecules may play a role in the chronic toxicity associated with formaldehyde exposure. Long-term occupational exposure to formaldehyde has been shown to have harmful effects on various organs, leading to carcinogenic, neurotoxic, reproductive, allergic, immunological, genetic, and respiratory health consequences (Bernardini et al., 2022).

#### 2.2.1 Carcinogenesis

An investigation involving over 25,000 formaldehyde-exposed workers across 10 plants established a significant association between formaldehyde exposure and the development of nasopharyngeal cancer, nasal and paranasal cancers, as well as leukemias (Kim et al., 2011). Based on these findings, the International Agency for Research on Cancer (IARC) classified formaldehyde as a human carcinogen (Group I), specifically for nasopharyngeal cancer in 2004 and for leukemia, particularly myeloid leukemia, in 2012 (Kim et al., 2011; Protano et al., 2021).

#### 2.2.2 Genotoxicity and mutagenicity

Formaldehyde has been shown to interact with DNA and exhibit genotoxicity in both in vitro and in vivo mutation tests (Kang et al., 2021). The National Institute for Occupational Safety and Health (NIOSH), the American Conference of Governmental and Industrial Hygienists (ACGIH), and the Occupational Safety and Health Administration (OSHA) all recommend that exposure to formaldehyde above established limits can adversely affect biomarkers of genotoxicity (Bernardini et al., 2022). These biomarkers include sister chromatid exchanges, DNA-protein cross-links, and micronucleus frequency, all of which are important indicators of carcinogenic potential. Key DNA alterations caused by formaldehyde exposure include DNA-protein cross-links and mutations in the phosphorylation of the tumor suppressor p53 (Umansky et al., 2022).

In a study of V79 Chinese hamster cells, formaldehyde induced DNA-protein cross-linking, sister chromatid exchanges, micronuclei formation, and cytotoxicity in a concentration-dependent manner (Kang et al., 2021). Human studies on occupational formaldehyde exposure have also demonstrated increased DNA damage, micronucleus formation, sister chromatid exchanges, and chromosome aberrations in peripheral lymphocytes and nasal mucosa (Kang et al., 2021). For example, sister chromatid exchange, a marker of DNA replication product interchanges between sister chromatids, was significantly elevated in peripheral blood lymphocytes from 57 pathologists with occupational exposure to formaldehyde (55.2 μg/m^3^) compared to controls (Ghelli et al., 2022). Additionally, rats exposed to inhaled formaldehyde (1, 30, and 300 ppb for 28 days) showed the presence of DNA mono adducts and DNA-protein cross-links in all tissues, further confirming the genotoxic effects of formaldehyde (Leng et al., 2019).

Formaldehyde has also been shown to induce mutagenicity. Gaseous exposure demonstrated direct, time-dependent, and dose-dependent mutagenic activity in five *Salmonella typhimurium* strains (TA98, TA100, TA1535, TA102, and TA1537) (Chen et al., 2020).

#### 2.2.3 Respiratory and lung toxicity

Epidemiological studies have demonstrated a significant association between formaldehyde exposure levels and the incidence and severity of various respiratory diseases. Formaldehyde induces inflammation in the respiratory tract through mechanisms such as oxidative stress, immune system activation, and airway remodeling. It also exacerbates pre-existing pulmonary inflammation and impairs lung function (Bhat et al., 2024). Formaldehyde damages respiratory epithelial cells, leading to loss of cellular function (Bernardini et al., 2022). For instance, exposure to formaldehyde has been shown to induce emphysema in rabbits. Histological analysis revealed ciliated shedding of nasal mucosal cells, vascular congestion, subepithelial edema, cell proliferation, and peribronchial lymphocyte infiltration (Squillacioti et al., 2021).

Formaldehyde exposure induces dose-dependent oxidative stress, which is detrimental to respiratory tissues and has a time-dependent carcinogenic effect on the upper respiratory tract. Notably, oxidative stress occurs even at occupational air-formaldehyde exposure levels below 0.10 ppm, the regulatory limit for air-formaldehyde exposure in occupational settings (Squillacioti et al., 2021).

#### 2.2.4 Neurotoxicity

Work-related exposure to formaldehyde has been associated with a range of neurological symptoms, including headaches, anxiety, fatigue, sleep disturbances, and cognitive impairments (Huang et al., 2019). Formaldehyde is also linked to neurodegenerative diseases, such as Alzheimer's disease, Parkinson's disease, and amyotrophic lateral sclerosis (Rana et al., 2021). Elevated levels of formaldehyde and increased expression of formaldehyde-generating enzymes have been reported in the brains of Alzheimer's patients (Tulpule & Dringen, 2013).

Formaldehyde affects neurological function through nonenzymatic condensation with neuramines, catecholamines, and indoleamines, resulting in the formation of tetrahydroisoquinoline and tetrahydro-beta carboline (THBC), respectively. In mice, THBC has been shown to impair cognitive function by causing the loss of passive avoidance retention and reducing spontaneous motor activity (Cao et al., 2021). Additionally, formaldehyde cross-links amyloid-beta (Aβ) monomers, leading to the formation of Aβ dimers, oligomers, and fibrils. These aggregation products induce neurotoxicity and are positively correlated with the severity of dementia in Alzheimer's disease patients (Kou et al., 2022).

Formaldehyde also impacts memory, learning, and behavior. The hippocampus, a region critical for learning and memory consolidation, has been shown to undergo increased cell apoptosis in the CA1 and CA3 regions of mice after formaldehyde exposure (Aydin et al., 2023), suggesting that formaldehyde disrupts memory consolidation.

#### 2.2.5 Reproductive and developmental toxicity

In male rats and mice, formaldehyde reduces sperm count, increases the percentage of deformed sperm cells, induces sperm malformation, raises the bone marrow micronucleus rate, damages testicular tissues, and decreases serum testosterone levels (Duong et al., 2011). Formaldehyde causes chromosome and DNA damage, oxidative stress, alterations in enzyme, hormone, and protein levels and functions, apoptosis, and toxicogenomic and epigenomic effects, all of which contribute to reproductive toxicity (Duong et al., 2011).

In female mice, formaldehyde leads to histological changes in the ovary and uterus, including irregular estrous cycles, smaller and damaged oocytes, reduced mitochondrial numbers, and fibrosis in reproductive tissues (Duong et al., 2011; Treesh et al., 2019). The expression of the Fas gene and increased caspase activity may play crucial roles in formaldehyde-induced ovarian toxicity in female animals. Notably, the expression of the Fas gene, caspase-8 mRNA, and the activities of caspase-8 and caspase-3 in ovarian tissue from formaldehyde-exposed animals were significantly higher than in controls and increased with dose (Peng et al., 2010).

In humans, occupational exposure to formaldehyde has been linked to increased menstrual disorders and infertility (Duong et al., 2011). Formaldehyde exposure also results in developmental toxicity, including spontaneous abortion, stillbirth, congenital malformations, low birth weight, and premature birth (Duong et al., 2011). A significant linear relationship has been observed, where higher serum formaldehyde concentrations correlate with an increased risk of miscarriage in 118 women compared to 191 healthy controls (Xu et al., 2017). Maternal exposure to formaldehyde during pregnancy increases the risk of spontaneous abortion and congenital heart malformations by 24 % (Treesh et al., 2019).

Moreover, formaldehyde has been associated with allergic reactions. Formaldehyde and its releasers (agents that release formaldehyde after usage) are commonly found in cosmetics, pharmaceuticals, household detergents, and industrial applications such as adhesives, paints, lacquers, and metalworking fluids (Søgaard et al., 2024). These compounds can induce contact allergies and allergic contact dermatitis. In the 1950s, the incidence of allergic reactions was as high as 3.9 % in Western Europe and North America, but this has since decreased to 1.5 %–2.5 % (Goossens & Aerts, 2022).

Despite its harmful effects, recent research has highlighted potential beneficial effects of formaldehyde. For instance, low concentrations of formaldehyde have been shown to promote the proliferation of human melanoma cells, activate the MAPK pathway, and support the survival of one‑carbon cycle-defective cells (Zhu et al., 2021).

## 3 Role of formaldehyde in the formation of harmful compounds in foods

In addition to its own toxicity, formaldehyde can be converted into various harmful compounds during food processing, including heterocyclic aromatic amines, methylimidazole, advanced glycation end products, N-nitrosamines, acrolein, and acrylamide.

### 3.1 Heterocyclic aromatic amines

**Fig. 1:**
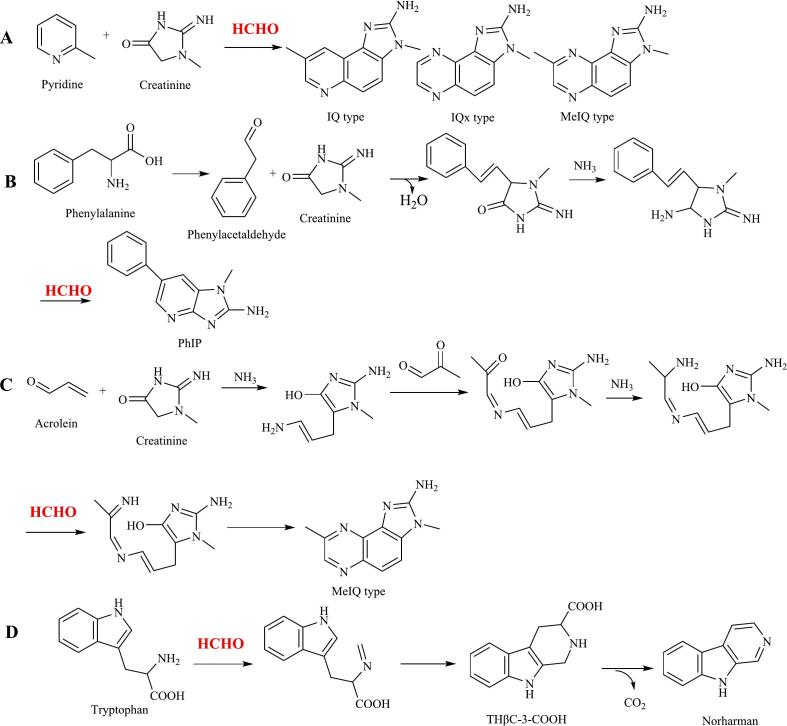
Role of formaldehyde in the formation of heterocyclic aromatic amines.

Heterocyclic aromatic amines (HAAs) are highly mutagenic and carcinogenic substances. These compounds typically feature a heterocyclic structure, often consisting of three aromatic rings with nitrogen atoms. HAAs can be divided into two categories: aminoimidazoazaarenes and amino carbolines, which include five-membered and six-membered heterocyclic amines (Dong et al., 2020). Four key types of heterocyclic aromatic amines are produced at higher levels under processing and cooking conditions (Hidalgo et al., 2021), including 2-amino-3-methylimidazo[4,5-*f*]quinoline (IQ), 2-amino-3,4-dimethylimidazo[4,5-*f*]quinoline (MeIQ), 2-amino-3,8-dimethylimidazo[4,5-*f*]quinoxaline (MeIQx), and 2-amino-1-methyl-6-phenylimidazo[4,5-*b*]pyridine (PhIP). All of these compounds contain an imidazole ring and either a pyridine or pyrazine ring (Fig. 1). Pyrazines are typically produced through Strecker degradation between α-dicarbonyl compounds and amino acids, while pyridines are thought to form via cyclization and oligomerization of short-chain reactive aldehydes with ammonia or ammonia-producing compounds (Zamora & Hidalgo, 2020).

The formation of three types of heterocyclic aromatic amines involves formaldehyde (Fig. 1). IQ, for example, is produced from creatinine (derived from creatine), which reacts with pyridine or pyrazine along with formaldehyde to generate IQ, IQx, and MeIQ (Fig. 1A). Formaldehyde also participates in the reaction between creatinine and phenylacetaldehyde (from phenylalanine) to form PhIP-type heterocyclic aromatic amines (Fig. 1B). However, the precise mechanisms underlying these processes remain unclear. Hidalgo and Zamora have proposed a more detailed formation pathway (Fig. 1C), which includes other reactive carbonyl compounds such as acrolein and methylglyoxal (Hidalgo & Zamora, 2021).

Additionally, formaldehyde is involved in the formation of norharman (Fig. 1D). In this pathway, formaldehyde reacts with tryptophan through the Pictet–Spengler reaction to generate tetrahydro-β-carbolines (THβCs) (Herraiz & Salgado, 2024). THβCs can form under moderate conditions, and concentrations of up to 500 mg/L have been detected in food products (Herraiz et al., 2023). Under thermal processing conditions, THβCs can be oxidized to form norharman (Chen et al., 2020).

### 3.2 4(5)-Methylimidazole and imidazole-type advanced glycation end products

Methylimidazoles, including 2-methylimidazole and 4(5)-methylimidazoles, are produced via the Maillard reaction in thermally processed foods and beverages (Hengel & Shibamoto, 2013). These compounds are classified as “possibly carcinogenic to humans” (Group 2B) by the International Agency for Research on Cancer (IARC) in 2011 and have been shown to induce hyperexcitation, convulsions, and anemia in animals (Akbari et al., 2023). Methylimidazoles also decrease sperm motility by disrupting the blood–testis barrier (Lu, Ma, et al., 2024). The precursor to methylimidazoles is methylglyoxal, a product of glucose degradation through the Maillard reaction and caramelization. Two pathways for the formation of methylimidazoles have been proposed, with formaldehyde playing a role in each.

**Fig. 2:**
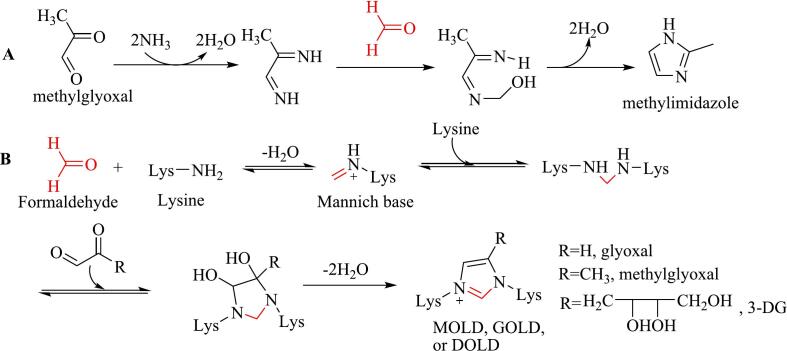
Role of formaldehyde in the formation of 4(5)-methylimidazole (A) and imidazole-type AGEs (B).

The first pathway is the ammonolysis reaction proposed by Moon and Shibamoto (2011). In this pathway, methylglyoxal undergoes ammonolysis to produce formamide, which then reacts with 2-aminopropanal (derived from the reaction of formaldehyde with acetamide) to generate 4- or 5-methylimidazole. The second pathway involves Debus–Radziszewski imidazole synthesis (Fig. 2A), where methylglyoxal reacts with ammonia to form an intermediate, which subsequently reacts with formaldehyde to produce methylimidazoles (Hengel & Shibamoto, 2013; Jang et al., 2013).

Dietary advanced glycation end products (dAGEs) are complex, heterogeneous compounds formed during nonenzymatic glycation reactions in food processing and cooking. These compounds are closely associated with various chronic diseases, including endogenous AGEs (Zhang et al., 2020). Key precursors of dAGEs include 1,2-dicarbonyl compounds such as glyoxal and methylglyoxal. These dicarbonyls react with lysine and arginine residues to produce various AGEs, including carboxymethyl lysine, imidazolium cross-link glyoxal lysine dimer (GOLD), carboxyethyl lysine, and methylglyoxal lysine dimer (MOLD) (Eggen & Glomb, 2021). Among these, GOLD, MOLD, and DOLD are formed with the participation of a C1 unit (Fig. 2B), exhibiting structures similar to those of methylimidazoles. Brinkmann et al. (1995) proposed a pathway for their formation, wherein glyoxal or methylglyoxal reacts with amino groups to form a diamine, which then reacts with another dicarbonyl compound. After oxidation and release of formic or acetic acid, followed by dehydration, an imidazolium end-product containing a C1 unit is formed.

Formaldehyde can be generated exogenously in foods and endogenously in humans (Hu et al., 2022; Li et al., 2023). Given that formaldehyde readily reacts with amino groups and dicarbonyl compounds at physiological temperatures to form imidazole salts (Li et al., 2023), we propose an alternative pathway for the generation of GOLD, MOLD, and DOLD, as shown in Fig. 2B. In this pathway, the ε-amino group of a residual lysine reacts with formaldehyde to form a Mannich base intermediate. Another lysine residue then reacts with the Mannich base to form an intermediate with two imino groups. Both imino groups attack the two carbonyl carbons of dicarbonyl compounds, leading to the dehydration of two water molecules and the formation of imidazoles.

### 3.3 N-nitrosamines

N-Nitrosamines are a class of mutagenic and carcinogenic compounds characterized by a nitroso functional group attached directly to a nitrogen atom. The lone pair of electrons on the amino nitrogen is delocalized into the π-electron system of the double-bonded oxygen, forming two major contributing resonance structures (López-Rodríguez et al., 2020). N-Nitrosamines are commonly found in drinking water, tobacco smoke, food, and household products (Li et al., 2024).

**Fig. 3:**
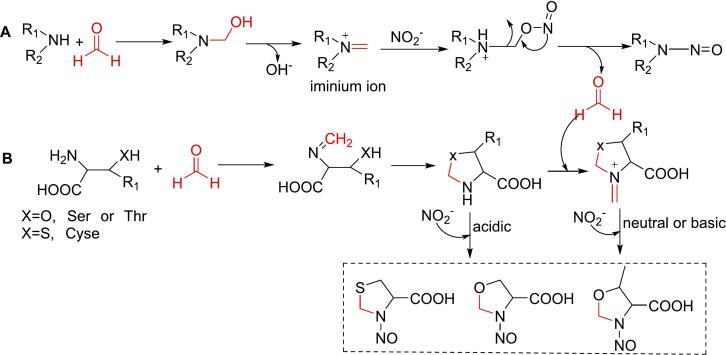
Role of formaldehyde in the formation of N-nitrosime from primary amines (A) and amino acids (B).

Nitrosation is most favorable under acidic conditions; however, under neutral and basic conditions, formaldehyde can efficiently catalyze the nitrosation of secondary amines to form N-nitrosamines (Ashworth et al., 2020; Eisenbrand et al., 2022). In this pathway, secondary amines react with formaldehyde to form an iminium ion intermediate, which then reacts with nitrite to yield a nitrosamine, with formaldehyde being eliminated (Fig. 3A). Formaldehyde significantly increases nitrosamine formation. In the presence of 0.05 M formaldehyde, nitrosamine formation from dimethylamine increased by 110 % at pH 3.0, 170 % at pH 3.5, 240 % at pH 4.0, 1700 % at pH 5.0, and 2000 % at pH 6.0 after 3.5 h of incubation at 37 °C (Kurechi et al., 1980).

The catalytic efficiency of formaldehyde is influenced by the type of amines. At pH 7, the presence of formaldehyde increased N-nitrosamine formation by factors of 26, 29, 10, 152, and 5 from dimethylamine, methylethylamine, diethylamine, pyrrolidine, and morpholine, respectively (Pan et al., 2024).

Moreover, formaldehyde can directly react with amino acids such as cysteine, serine, and threonine to generate N-nitrosamines. In this process, formaldehyde reacts with the amino group to form a Mannich base, which subsequently reacts with the SH group in cysteine or the OH group in serine or threonine to generate heterocyclic carboxylic acids (Kamps et al., 2019). These heterocyclic carboxylic acids are readily nitrosatable under acidic conditions, reacting with nitrite to produce various N-nitroso compounds (Tricker & Kubacki, 1992). Rather than acting as a catalyst, formaldehyde participates in N-nitrosamine formation (Fig. 3B). Under neutral or basic conditions, we hypothesize that the imino groups in the heterocyclic carboxylic acids may further react with formaldehyde to form iminium ions, which then react with nitrite to generate N-nitrosamines (Fig. 3B). Given that formaldehyde significantly enhances the formation of N-nitrosamines (Kurechi et al., 1980; Pan et al., 2024), this mechanism may contribute to the generation of large quantities of N-nitrosamines from amino acids during the thermal processing of foods.

Additionally, 1, 2-dicarbonyl compounds, which are commonly present in foods, have been reported to generate formaldehyde, particularly under basic conditions (Li et al., 2023). Therefore, typical dicarbonyl compounds, such as glyoxal, methylglyoxal, and 1-deoxyosone, may also promote N-nitrosamine formation in the presence of nitrite.

### 3.4 Cross-linking of proteins and nucleic acids (cross-linking adducts)

**Fig. 4:**
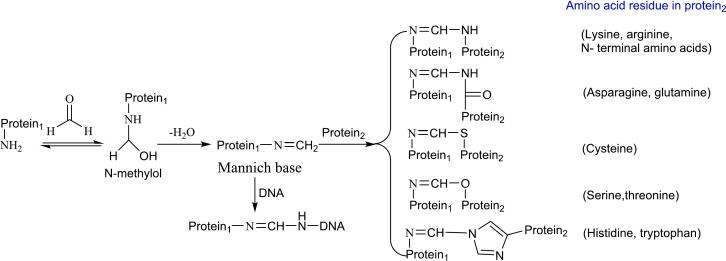
Cross-linking between proteins as well as between proteins and nucleic acids by formaldehyde.

Formaldehyde is susceptible to attack by SH groups, amino groups in proteins and in nucleic acid bases (Jia et al., 2024). It acts as an efficient cross-linking agent between proteins, as well as between proteins and nucleic acids (Piersimoni et al., 2021). During the cross-linking process, formaldehyde undergoes nucleophilic addition reactions with amine groups (such as lysine residue) in proteins to yield *N*-methylol, which is then dehydrated and condensed into a Schiff base (Mannich base). The Mannich base is a highly electrophilic intermediate that can react with amino acids containing amino, imino, amide, sulfhydryl, and hydroxyl groups in the same protein (intramolecular cross-linking) or another protein (intermolecular cross-linking), resulting in protein cross-linking (Fig. 4). The Mannich base can also react with the amino group in DNA or RNA, leading to protein-nucleic acid cross-linking. Formaldehyde can also cross-link proteins through the dimerization of two imine-modified lysines that are spatially close (Tayri-Wilk et al., 2020).

The cross-linking between proteins or nucleic acids hinders gene transcription and disrupts cell proliferation (Jia et al., 2024), and has detrimental effects on human health. Recent reports indicate that formaldehyde-induced cross-linking is associated with the progression of various diseases, including cardiovascular disease, Alzheimer's disease, Parkinson's disease, amyotrophic lateral sclerosis, and brain cancer (Rana et al., 2021; Zhang, Yang, et al., 2021; Zhao et al., 2021).

Protein-formaldehyde cross-linking adducts exhibit cytotoxicity. Thiazolidine-4-carboxylic acid, a thiazolidine adduct formed between formaldehyde and cysteine residues, shows significant toxic effects on proteins and cells (Patterson et al., 2020). The globular-like aggregates of Tau, a Tau-formaldehyde adduct, cause oxidative stress and cell apoptosis in SH-SY5Y cells and rat hippocampal cells (He & He, 2017). The cross-linking adducts of Tau may play a role in Tauopathies. However, moderate, limited cross-linking may have a beneficial role. Recently, Zhu et al. (2021) found that cross-linking between residues Cys11 and Lys13 on the formaldehyde-responsive transcription factor HxlR by formaldehyde enhanced its DNA affinity and transcriptional activation.

In addition to the aforementioned harmful compounds, formaldehyde can be converted into acrolein, a highly toxic agent associated with the development of various diseases, including cardiovascular disease, alcoholic liver disease, Alzheimer's disease, diabetes, aging, and chronic obstructive pulmonary disease (Jiang et al., 2022; Wang et al., 2023). The cross-condensation of formaldehyde and acetaldehyde produces acrolein, which is readily formed and was the first commercial synthesis method for acrolein, discovered in 1942 (Folliard et al., 2020). In thermally processed foods, acrolein can be further converted into acrylic acid and subsequently generate acrylamide. Acrylamide has been classified as a priority control contaminant by the U.S. Environmental Protection Agency and as a Group 2 A carcinogen by the International Agency for Research on Cancer (IARC). It is associated with neurotoxicity, genotoxicity, reproductive toxicity, developmental toxicity, and carcinogenicity (Faraji et al., 2024). The Commission Regulation (EU) 2017/2158 has set benchmark levels for acrylamide in certain foods (Rampazzo et al., 2024).

## 4 Role of formaldehyde in the formation of flavor compounds in foods

Although formaldehyde is a toxic contaminant that generates harmful derivative compounds in foods, it also contributes to flavor formation, particularly in the development of heterocyclic aromas. In this context, we focus on furanones, pyrazines, and thiazolidines as examples of formaldehyde-derived compounds in oxygen-, sulfur-, and nitrogen-containing heterocyclic aromas. The occurrence and the odor threshold of the main aromas were listed in Table 2.

**Table 2:** Occurrence and odor threshold of typical aromas formed by the participation of formaldehyde.

Compounds	Odor threshold	Occurrence	References
4-Hydroxy-2,5-dimethyl-3(2H)-furanone (HDMF)	60 μg/kg	Fruits, baked foods, ice wines, cooked meat, baked foods with a fruity, sweet, and caramel aroma flavor.	Xiao et al., 2021
5-Methylfuran-2-carbaldehyde	2400 mg/kg	Vinegar, roasted Arabica coffee with a sweet, caramel-like aroma	Al-Dalali et al., 2020; Cai et al., 2024
2,3,5-Trimethylpyrazine	9 mg/kg	They occur ubiquitously in nature and almost all heated foods. Mainly contribute to roasted or toasted flavors of cooked foods.	Liang et al., 2022; Koehler et al., 1971; Müller & Rappert, 2010
Tetramethylpyrazine	10 mg/kg		
2, 5-Dimethylpyrazine	35 mg/kg		
2, 6-Dimethylpyrazine	54 mg/kg		
2-Methylpyrazine	105 mg/kg		
2,3-Dimethylthiophene	5 mg/kg	Vegetable (garlic, onion, broccoli, cabbage, cauliflower), and thermally-processed foods (roasted meat, chicken, seafood, and coffee).	Galetto & Hoffman, 1976; McGorrin, 2011
2,4-Dimethylthiophene	3 mg/kg		
2,5-Dimethylthiophene	3 mg/kg		
3,4-Dimethylthiophene	5 mg/kg		
3,5-Dimethyl-1,2,4-trithiolane	10 μg/kg	In durian with strong sulfury onion-like and delicately fruity.	Buettner, 2017
2,5-Dimethylthiazole	8.9 × 10^−11^ mM	Orange juice.	Dreher et al., 2003
3,4-hexanedione	5 mg/kg	In baked goods with buttery, cooked, and caramel-like flavor.	Anderson et al., 2013
2-hydroxy-3(2H)-thiophenones	10 μg/kg	In cooked beef, coffee, roasted filberts and roasted peanuts.	Wang et al., 2024
2,5-dimethyl-4-hydroxy-3-(2H)-thiophenone	1.6 ng/L	Beef, roasted meat flavor.	Hofmann & Schieberle, 1997
2,5-dimethyl-2,4-dihydroxy-3(2H)-thophenone	1 mg/kg	Soy sauce, beer, roasted wheat grains, bread, burnt garlic, onion and miso.	Chukwu et al., 2017; Furusawa et al., 2013
1-(2-furanyl)-ethanone	No data available	In fish sauce, cooked oysters, dry-cured beef, and roasted fish with nutty aroma.	Yu et al., 2023
vinylpyrazine	0.7 mg/kg	Coffee, cocoa, soy sauce, wine, and liquor.	Gemert, 2011; Ma et al., 2022
5-methyl-2-thiophene	1.75–7.4 μg/m^3^	Roasted peanuts.	Gemert, 2011; McGorrin, 2012
2-formyl-5-methylthiophene	0.032 μg/m^3^	Baked flaxseed.	Gemert, 2011; Ni et al., 2022
2-methylthiazole	2–20 μg/kg	Ham, beef, corn, sesame.	Yeo et al., 2022 Martínez-Onandi et al., 2016; Puvipirom & Chaiseri, 2012
1,2,3,5,6-pentathiepane	0.27–0.53 mg/kg	Dried mushroom.	Gemert, 2011; Schmidberger & Schieberle, 2020
1,2,3-trithiolane	7.66 μg/kg	Cooked allium.	Gemert, 2011; Flaig & Granvogl, 2015

### 4.1 Furanones

**Fig. 5:**
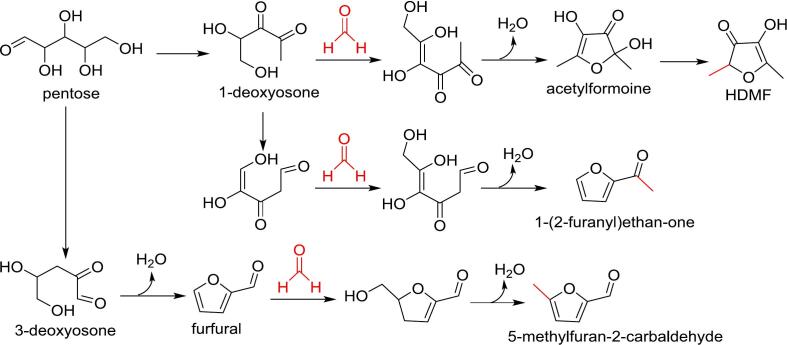
Role of formaldehyde in the formation of furanones and methylfuran-2-carbaldehyde.

Furanones are five-membered heterocycles that play a significant role in the formation of food flavors. Key furanones include 4-hydroxy-2,5-dimethyl-3(2H)-furanone (HDMF), 5-ethyl-4-hydroxy-2-methyl-3(2H)-furanone, and 5-methyl-4-hydroxy-3(2H)-furanone (Xiao et al., 2021). Among these, HDMF (also known as furaneol), which has very low odor threshold (Table 2) and exhibits a caramel-like, sweet, and fruity aroma, is a prominent flavor compound contributing to the sensory properties of a variety of natural and processed foods, such as pineapple, tomato, grape, roasted coffee, roasted almond, baked goods, beef broth, roasted beef, stewed beef, and soy sauce (Blank & Fay, 1996; Wang et al., 2009). The formation of HDMF is illustrated in Fig. 5. Under thermal conditions, pentose sugars (xylose and arabinose) undergo degradation to form 1-deoxyosone, which reacts with formaldehyde through aldol-type condensation to generate a six‑carbon intermediate. This intermediate then undergoes enolization and dehydration, leading to the formation of a cyclic acetylated intermediate, which ultimately results in HDMF following reduction, enolization, and water elimination (Blank & Fay, 1996). Other aromas, including 3,4-hexanedione, 2-hydroxy-3(2H)-thiophenones, 2,5-dimethyl-4-hydroxy-3-(2H)-thiophenone, 2,5-dimethyl-2,4-dihydroxy-3(2H)-thiophenone, have been reported to be derive from the reaction of HDMF with cystine (Xiao et al., 2021).

Additionally, chain elongation of pentose degradation products by formaldehyde leads to the formation of other furan-derived flavor compounds. For instance, 1-(2-furanyl)-ethanone, a flavor compound found in fish sauce, cooked oysters, dry-cured beef, and roasted fish (Hwang et al., 2015; Lu, Lin, & Fang, 2024; Peralta et al., 1996; Sha et al., 2017), is also derived from 1-deoxyosone. Following isomerization and an aldol condensation reaction with formaldehyde, the subsequent dehydration of the intermediate leads to the formation of 1-(2-furanyl)-ethanone (Fig. 5) (Bernardini et al., 2022). Furthermore, the dehydration product of furfural can react with formaldehyde to generate 5-methylfuran-2-carbaldehyde (Zhan et al., 2022), a compound with a sweet, caramel-like aroma (Cai et al., 2024).

### 4.2 Pyrazines

Pyrazines are utilized as flavoring agents and preservatives in food products, possessing a core structure of six-membered heterocycles with nitrogen atoms located at the para-positions. Naturally occurring pyrazines can be substituted at the 2-, 3-, 5-, or 6-positions with various functional groups, including alkyl (primarily methyl and ethyl), methoxyl, acyl, and sulfur-containing groups such as thiols or sulfides (Fayek et al., 2023; Zhao et al., 2020). Pyrazines primarily form during the heating of foods and are commonly present in coffee, cereal products, meat products, and wine (Ren et al., 2024).

**Fig. 6:**
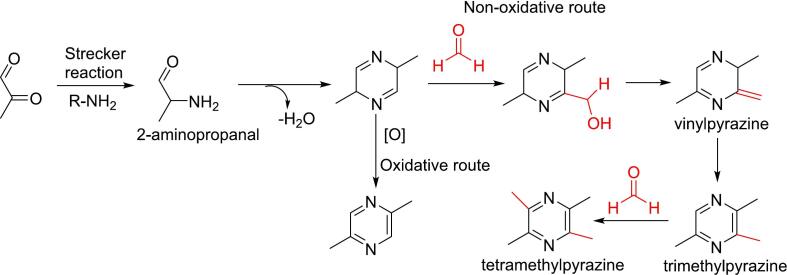
Role of formaldehyde in the formation of pyrazine via Strecker degradation between methylglyoxal and amino acids.

Pyrazine formation is primarily driven by two mechanisms: the Strecker degradation and the ammonia/acyloin reaction. During thermal processing, pyrazines arise from dicarbonyl precursors produced in the Maillard reaction. These dicarbonyl compounds undergo Strecker degradation with amino acids, forming unstable Schiff bases that subsequently decarboxylate and hydrolyze to yield α-aminocarbonyls (aminoketones or amino aldehydes). These intermediates then condense to produce dihydropyrazines, which can either oxidize to stable pyrazines or react with carbonyl or aldehyde groups, forming alkylpyrazines via a nonoxidative route (Fig. 6) (Poisson et al., 2009; Zhao et al., 2020). In addition to proline, pyrazines can be synthesized from other 19 amino acids through the Strecker pathway (Xing & Yaylayan, 2024).

Alternatively, pyrazines may form via the ammonia/acyloin reaction, where acyloin (α-hydroxy ketone) condenses with ammonia to yield α-amino ketones, which further condense to produce dihydropyrazine. This dihydropyrazine can proceed through oxidative or nonoxidative routes to form pyrazines (Shu & Lawrence, 1995). Acyloin, produced by pyruvate decarboxylase, is a secondary metabolite found in many fungi, plants, and bacteria (Rizzacasa & Ricca, 2023), making this pathway specific to pyrazine synthesis in biological organisms.

Formaldehyde plays a role in modifying the pyrazine structure. Initially, it integrates into the side chain of dihydropyrazine to form substituted pyrazines, such as vinylpyrazines, 2,3,5-trimethylpyrazine, and tetramethylpyrazines (Fig. 6) (Low et al., 2007; Ma et al., 2022; Poisson et al., 2009). This transformation follows the nonoxidative pathway of pyrazine formation (Parker et al., 2010; Zhan et al., 2022). When glyoxal is used as the precursor, formaldehyde can attach to each carbon in the dihydropyrazine cycle (Low et al., 2007). Additionally, formaldehyde extends the carbon chains of dicarbonyl compounds involved in pyrazine synthesis. For instance, 2, 3, 5-trimethylpyrazine forms from glyoxal and amino acids in the presence of formaldehyde through methylglyoxal conversion. Formaldehyde reacts with glycolaldehyde from glyoxal reduction, yielding 2,3-dihydroxypropanal, which dehydrates to 2-hydroxyacrylaldehyde and subsequently converts to methylglyoxal (Jiang et al., 2023). In pet food, formaldehyde significantly enhances the formation of trimethylpyrazine and other pyrazines derived from 2,3-butanedione, which itself is generated from the aldol condensation of methylglyoxal and formaldehyde (Parker et al., 2010).

Formaldehyde plays important role in elevation of odor threshold of pyrazine via the methylization. As shown in Table 2, the pyrazines with three or four methyl-substituted (2,3,5-trimethylpyrazine, tetramethylpyrazine) display 5- to 10-fold lower threshold than one methyl-substituted pyrazine (2-methylpyrazine).

### 4.3 Thiazoles

Thiazoles are five-membered aromatic rings containing sulfur and nitrogen. The most prominent types of thiazoles include alkylthiazoles, acetylthiazoles, and hydroxyethylthiazoles. Alkylthiazoles, such as 2,4,5-trimethylthiazole, are commonly found in foods like meat, potatoes, coffee, cooked beef, roasted lamb, and cooked chicken. These compounds impart a chocolate, nutty, coffee-like aroma, along with a meaty flavor (Rowe, 2009).

**Fig. 7:**
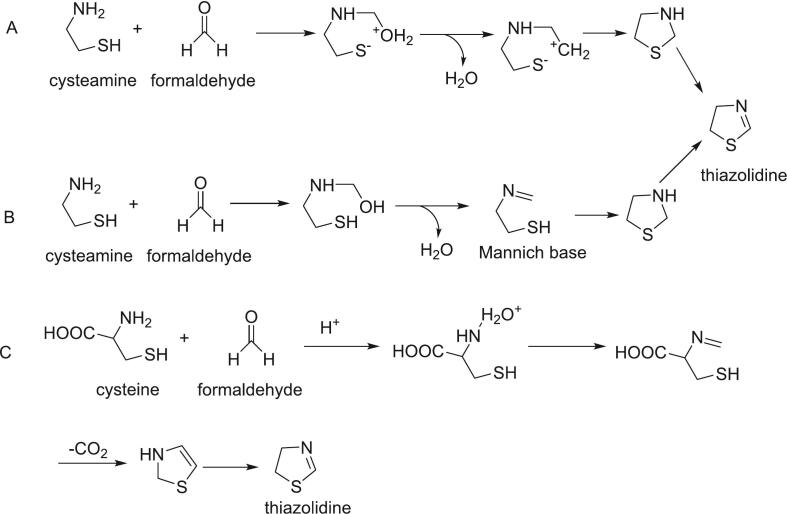
Role of formaldehyde in the formation of thiazolines.

Sakaguchi and Shibamoto (1978) reported the detection of thiazolidine in the reaction mixture of d-glucose and cysteamine, which is generated from the reaction of glyoxal with cysteine (Liu et al., 2022). In this reaction, the amine group of cysteamine attacks the carbonyl group of formaldehyde. Protonation of the hydroxyl group leads to the formation of a water molecule and a carbocation. A subsequent nucleophilic attack by the thiolate on the carbocation forms a substituted thiazolidine (Fig. 7A). Cysteamine can also react with formaldehyde to produce a Mannich intermediate, which then attacks the thiol group (SH), forming a five-membered ring that undergoes dehydrogenation to produce thiazolidine (Fig. 7B). Du et al. (2024) proposed that cysteine can directly react with formaldehyde to form thiazolidine via a similar pathway. In this case, the amine of cysteine undergoes a nucleophilic attack on formaldehyde, followed by cyclization of the Mannich base, decarboxylation, and oxidation (dehydrogenation) to produce thiazolidine (Fig. 7C).

In addition to thiazolidine, formaldehyde participates in the formation of other sulfur-containing cyclic aromas, such as thiophene, thiazoles, and polysulfides. For example, compounds like 5-methyl-2-thiophene, 2-formyl-5-methylthiophene, 2-methylthiazole, and 2,5-dimethylthiazole are derived from formaldehyde (Fan et al., 2023; Zhai et al., 2023). Furthermore, formaldehyde readily reacts with hydrogen sulfide to generate various polysulfides, including 3,5-dimethyl-1,2,4-trithiolane, 1,2,3,5,6-pentathiepane, 4,6-dimethyl-1,2,3-trithiane, and 1,2,3-trithiolane (Feng et al., 2024). These sulfur-containing compounds, with their low aroma thresholds (Table 2), significantly contribute to the flavor profile of foods.

## 5 Conclusion and future prospects

Formaldehyde is detected at very low levels in processed foods, although it may be present in higher concentrations in food materials and can be generated during food processing. This is due to its high reactivity, which leads to its conversion into various other compounds. This review focuses on two primary aspects of formaldehyde conversion: the formation of formaldehyde-derived toxins and aromas.

Future research should focus on three key areas. First, the precise formation mechanisms of formaldehyde during food processing need to be elucidated, and the potential or actual formation levels of formaldehyde in thermally processed foods should be estimated. Second, in-depth investigation of the transformation pathways of formaldehyde into other compounds, particularly its elimination routes during food processing, is necessary. Third, strategies to inhibit formaldehyde formation should be explored, and efforts should be directed toward redirecting the formation pathway of harmful compounds toward the production of beneficial compounds.

## CRediT authorship contribution statement

**Xiaoyan Sun:** Writing – original draft. **Chunmin Yang:** Writing – review & editing. **Weiyue Zhang:** Writing – review & editing. **Jie Zheng:** Methodology, Investigation. **Juanying Ou:** Resources, Methodology. **Shiyi Ou:** Supervision, Funding acquisition, Conceptualization.

## Declaration of competing interest

The authors declare that they have no known competing financial interests or personal relationships that could have appeared to influence the work reported in this paper.

## Data Availability

No data was used for the research described in the article.
